# Clinical and Cognitive Correlates of Amyloid‐Related Imaging Abnormalities During Lecanemab Treatment in Early Alzheimer's Disease

**DOI:** 10.1002/alz70861_109016

**Published:** 2025-12-23

**Authors:** Nicole Veloro Tayag, Madelaine Lowery, Neel Edupuganti

**Affiliations:** ^1^ Medical College of Georgia, Augusta, GA USA

## Abstract

**Background:**

Lecanemab, a monoclonal antibody targeting amyloid‐β protofibrils, has demonstrated efficacy in reducing amyloid plaque burden and slowing cognitive decline in early Alzheimer’s disease (AD). However, treatment is associated with amyloid‐related imaging abnormalities (ARIA), including vasogenic edema (ARIA‐E) and cerebral microhemorrhages or superficial siderosis (ARIA‐H). The cognitive consequences of ARIA remain insufficiently characterized in real‐world settings.

**Method:**

This retrospective cohort study included 92 individuals with mild cognitive impairment or mild AD who received Lecanemab between 2023 and 2025 at a single community memory clinic. All patients had biomarker‐confirmed amyloid pathology via plasma pTau217 or amyloid PET imaging. Clinical data extracted included demographics, vascular risk factors, APOE4 carrier status, antithrombotic use, infusion number, ARIA subtype and severity, and serial MRI findings. Cognitive performance was assessed longitudinally using the Mini‐Mental State Examination (MMSE) and Montreal Cognitive Assessment (MoCA). The primary objective was to evaluate associations between ARIA characteristics and cognitive trajectories.

**Result:**

The majority of patients (n = 58, 63%) did not develop ARIA. Among ARIA‐positive patients (n = 34, 37%), isolated ARIA‐H and combined ARIA‐H with ARIA‐E were the most prevalent subtypes. ARIA‐E resolved in 63% of cases on follow‐up imaging, whereas ARIA‐H persisted in 60%. Most ARIA events (71%) were asymptomatic and mild to moderate in severity. There was no significant difference in cognitive outcomes between patients with asymptomatic ARIA and those without ARIA (mean MMSE 28.1 vs. 28.5, *p* = 0.34). However, patients with symptomatic ARIA demonstrated significantly lower follow‐up MMSE scores compared to both asymptomatic ARIA and ARIA‐negative individuals (mean MMSE 25.0, *p* = 0.004 and *p* = 0.02, respectively). A non‐significant trend toward lower cognitive scores was observed in those with higher ARIA‐H burden.

**Conclusion:**

In this real‐world cohort, symptomatic ARIA—particularly ARIA‐H—was associated with measurable cognitive decline. While most ARIA cases were asymptomatic and did not impact cognition, these findings highlight the need for routine neuroimaging surveillance and individualized treatment strategies to optimize safety and cognitive outcomes during Lecanemab therapy. This highlights the need for ongoing assessment of ARIA in clinical practice to enhance the safety and efficacy of amyloid‐targeting therapies in early AD.

